# Innovative Self-Cleaning and Biocompatible Polyester Textiles Nano-Decorated with Fe–N-Doped Titanium Dioxide

**DOI:** 10.3390/nano6110214

**Published:** 2016-11-15

**Authors:** Ionela Cristina Nica, Miruna Silvia Stan, Anca Dinischiotu, Marcela Popa, Mariana Carmen Chifiriuc, Veronica Lazar, Gratiela G. Pircalabioru, Eugenia Bezirtzoglou, Ovidiu G. Iordache, Elena Varzaru, Iuliana Dumitrescu, Marcel Feder, Florin Vasiliu, Ionel Mercioniu, Lucian Diamandescu

**Affiliations:** 1 Department of Biochemistry and Molecular Biology, Faculty of Biology, University of Bucharest, 91-95 Splaiul Independentei, 050095 Bucharest, Romania; cristinai.nica@gmail.com (I.C.N.); miruna.stan@bio.unibuc.ro (M.S.S.); 2 Department of Botanic-Microbiology, Faculty of Biology, University of Bucharest, 1-3 Aleea Portocalelor, 60101 Bucharest, Romania; bmarcelica@yahoo.com (M.P.); veronica.lazar2009@gmail.com (V.L.); 3 Research Institute of the University of Bucharest—ICUB, University of Bucharest, 91-95 Splaiul Independentei, 050095 Bucharest, Romania; gratiela87@gmail.com; 4 Department of Agricultural Development, Democritus University of Thrace, 67100 Xanthi, Greece; empezirt@agro.duth.gr; 5 National R & D Institute for Textiles and Leather Bucharest (INCDTP), 16 Lucretiu Patrascanu, 030508 Bucharest, Romania; iordacheovidiu.g@certex.ro (O.G.I.); elena.varzaru@certex.ro (E.V.); iuliana.dumitrescu@certex.ro (I.D.); 6 National Institute of Materials Physics (NIMP), Atomistilor 405A, 077125 Bucharest-Magurele, Romania; mfeder@infim.ro (M.F.); fvasiliu@infim.ro (F.V.); imercioniu@infim.ro (I.M.); diamand@infim.ro (L.D.)

**Keywords:** photocatalyst, titanium dioxide, polyester textile, antibacterial, skin fibroblasts

## Abstract

The development of innovative technologies to modify natural textiles holds an important impact for medical applications, including the prevention of contamination with microorganisms, particularly in the hospital environment. In our study, Fe and N co-doped TiO_2_ nanoparticles have been obtained via the hydrothermal route, at moderate temperature, followed by short thermal annealing at 400 °C. These particles were used to impregnate polyester (PES) materials which have been evaluated for their morphology, photocatalytic performance, antimicrobial activity against bacterial reference strains, and in vitro biocompatibility on human skin fibroblasts. Microscopic examination and quantitative assays have been used to evaluate the cellular morphology and viability, cell membrane integrity, and inflammatory response. All treated PES materials specifically inhibited the growth of Gram-negative bacilli strains after 15 min of contact, being particularly active against *Pseudomonas aeruginosa*. PES fabrics treated with photocatalysts did not affect cell membrane integrity nor induce inflammatory processes, proving good biocompatibility. These results demonstrate that the treatment of PES materials with TiO_2_-1% Fe–N particles could provide novel biocompatible fabrics with short term protection against microbial colonization, demonstrating their potential for the development of innovative textiles that could be used in biomedical applications for preventing patients’ accidental contamination with microorganisms from the hospital environment.

## 1. Introduction

Over the past decade, the emergence and spread of an increasing number of antibiotic-resistant microorganisms has become a serious worldwide concern [1,2]. As all existing natural and chemical agents have lost their efficiency, the search for new methods of sterilization is vital [3]. Photocatalysis has become one of the most effective of all modern techniques because it leads to complete mineralization of organic contaminants, including pathogens [4].

The photocatalytic process has been extensively studied using titanium dioxide (TiO_2_), an ideal material in terms of chemical properties and economical aspect, but having the major disadvantage of radiation’s absorption only in the ultraviolet region [5]. For this reason, TiO_2_ nanoparticles (TiO_2_ NPs) were doped with other metals (Ag, Cu, Fe, Zn, Zr) [6], non-metals (C, N), combinations of metals and non-metals, or metals and rare earth elements [7] to extend the photocatalytic activity of TiO_2_ in visible light, thereby, to obtain increased efficiency of pollution agents’ degradation, including pathogens [8,9].

Smart materials with improved features, obtained through the deposition of photoactive nanoparticles on fabrics [10], have gained increasingly more interest on the market. In addition to multifunctional properties, a valuable product should fulfill economic and environmental requirements [11]. The development of innovative technology in order to modify natural materials can be a cheaper alternative than using expensive fibers that already have incorporated performance properties [12]. Usually, new properties can be added to textiles by incorporating functional agents onto their fibers using chemical or physical procedures [13], without any color changes [14]. Noble metal and metallic oxide NPs are the most used to confer antibacterial (against *Escherichia coli*, *Staphylococcus aureus*, and *Klebsiella pneumoniae*) [15,16] and antifungal (against *Aspergillus niger* and *Candida albicans*) activities [17,18]. In addition to these, the photocatalysts were intensively investigated to develop multifunctional textiles with special properties, such as self-cleaning and stain repellence [19,20,21], ultraviolet (UV) and electromagnetic radiation (EMR) shielding [22], hydrophobic-hydrophilic [23], wrinkle-freeness, static elimination, and electrical conductivity [24,25,26]. Consequently, different photocatalytic fabrics based on cotton, polyester, wool, polyamide, or cellulose fibers were designed in previous years [27,28]. These fabrics with enhanced functionalities are of great interest for medical environments, textile and food industries due to their ability to inhibit the formation of pathogen biofilms, stop the spread of nosocomial infections, and remove the infection sources [29].

In this study, polyester (PES) fabrics (142 g/m^2^, 0.295 mm thickness) were treated with TiO_2_ particles co-doped with 1% iron and nitrogen atoms (TiO_2_-1% Fe–N) according to the methods described in our previous work [28]. The Fe–N doping was chosen due to their physical particularities: nitrogen is able to extend the absorption in the visible region and iron can reduce the electron-hole recombination [30,31]. Depending on the phase composition and band gap, the photodegradation efficiency can be improved by 75% under visible light [32]. In a first step, the fabrics were immersed in a fresh particle suspension and, after that, new fabrics were soaked in the remaining dispersion from the first treatment and, consecutively, a polyacrylic binder was used to fix the particles. Using this approach, the amount of doped TiO_2_ particles which were released into the environment and the water, and the chemicals’ consumption, were reduced [28].

Scanning electron microscopy (SEM) and energy dispersive X-ray spectroscopy (EDX) analysis were used to characterize the morphology of the coated fibers and the nanoparticles’ distribution on the fibers. The photocatalytic performance and self-cleaning efficiency of TiO_2_ NPs deposited on PES fabrics was evaluated by observing the changes in color parameters after staining with methylene blue (MB) or coffee, and exposure to visible and ultraviolet light. In addition, the antimicrobial activity was tested against Gram-negative (*E. coli*, *Pseudomonas aeruginosa*) bacterial reference strains using standardized quantitative assays. Finally, biocompatibility was assessed on CCD-1070Sk normal human dermal fibroblasts by cellular morphology, viability, and cell membrane integrity analyses after 6 and 12 h of exposure to the NP-treated fabrics to obtain novel and valuable experimental data for further in vivo purposes.

## 2. Results

### 2.1. Characterization of Photocatalysts

X-ray diffraction (XRD), X-ray photoelectron spectroscopy (XPS), transmission electron microscopy (TEM) and Mössbauer spectroscopy were used to study the structure and morphology of the obtained nanoscaled photocatalysts.

**Figure 1:**
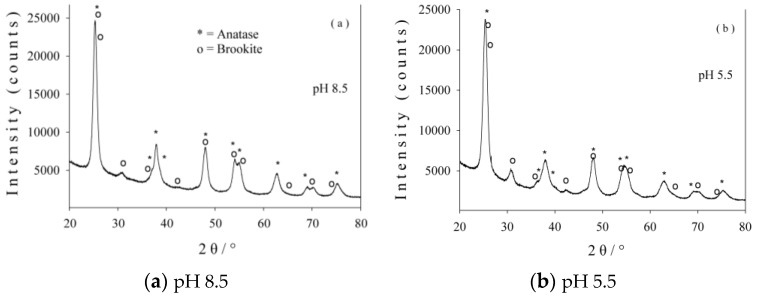
X-ray diffraction (XRD) patterns of photocatalyst 1 (**a**) and photocatalyst 2 (**b**).

In Figure 1 the XRD patterns of both photocatalysts are depicted. Rietveld refinements revealed the presence of a prevailing anatase phase accompanied by a brookite phase in both samples. The crystallite sizes (given by Scherrer equation [33]) are in the range of 10–12 nm for the anatase phase and 8–11 nm for the brookite phase, respectively (Table 1). XPS and Mössbauer spectroscopy certified the presence of nitrogen and Fe^3+^ in the obtained samples. The photocatalytic tests, in both UV and visible spectral regions, were performed by means of a PCC-2 (Ulvac-Riko Inc., Yokohama, Japan & ULVAC GmbH, Ismaning, Germany) photocatalytic checker. A detailed analysis was presented in a previous paper [28].

**Figure 2:**
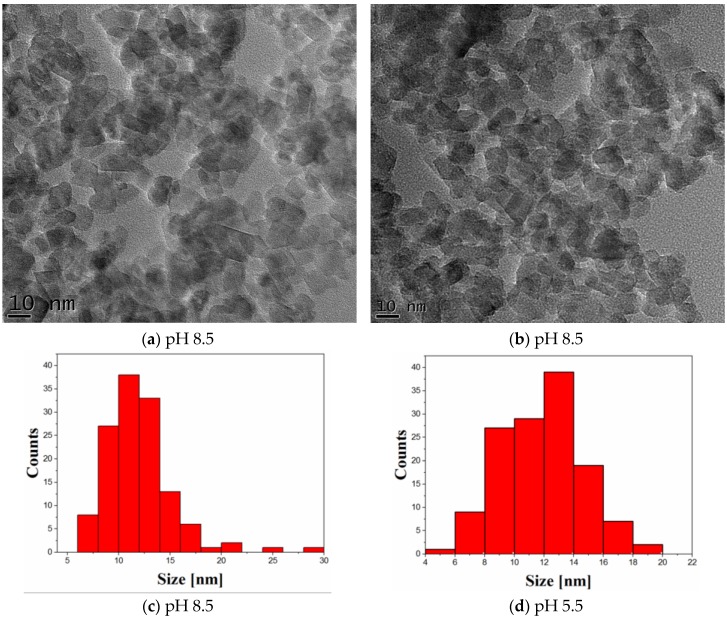
Transmission electron microscopy (TEM) images (**a**,**b**) of TiO_2_-1% Fe–N photocatalysts and their corresponding size distribution (**c**,**d**) for: pH 8.5 (photocatalyst 1) and pH 5.5 (photocatalyst 2).

TEM images (Figure 2a,b) display the powder morphology of nanoscaled photocatalysts obtained by hydrothermal treatment and co-precipitation at different pH values corresponding to pH 8.5 (photocatalyst 1) and pH 5.5 (photocatalyst 2). A predominant quadratic morphology can be observed in the first case, and a combined morphology (triangular, spherical, etc.) can be noticed for the second photocatalyst. Furthermore, the particle distribution was evaluated for the photocatalysts 1 and 2, as displayed in Figure 2c,d, respectively. The statistical measurements were performed on more than 100 particles. The mean size of ~15 nm with standard deviation ~2.8 nm corresponding to pH 5.5 decreased to ~10 nm with standard deviation, and ~4 nm in the case of pH 8.5, in rather good agreement with XRD results (Figure 1).

### 2.2. SEM/EDX Analysis of the Treated Fabrics

**Figure 3:**
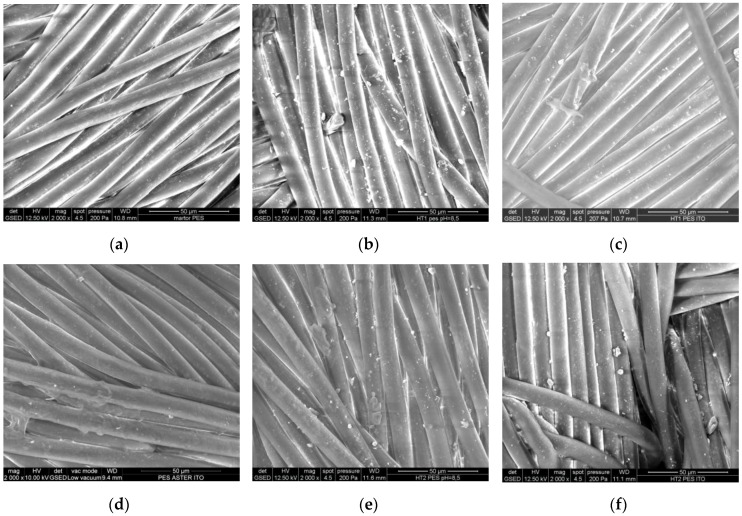
Scanning electron microscopy (SEM) images of polyester fabrics: Control-polyester (PES) fabric (**a**); HT1-PES fabric treated with TiO_2_-1% Fe–N (photocatalyst 1) (**b**); HT1 polyacrylic binder (ITO)-PES fabric treated with TiO_2_-1% Fe–N (photocatalyst 1) and ITO (**c**); PES ITO-PES fabric treated with polyacrylic binder (ITO) (**d**); HT2-PES fabric treated with TiO_2_-1% Fe–N (photocatalyst 2) (**e**); HT2 ITO-PES fabric treated with TiO_2_-1% Fe–N (photocatalyst 2) and ITO (**f**).

Scanning electron microscopy (SEM) analysis (Figure 3) showed the modification of the polyester fibers, which are non-uniformly coated by doped TiO_2_ particles. Regardless of the photocatalyst used, most of the particles form clusters. Their sizes varied slightly higher in the case of treatment with the photocatalyst 2 (270–288 nm) compared to photocatalyst 1 (132.6–278 nm). The polyacrylic binder (ITOBINDER AG, abbreviated ITO) induced a higher aggregation of the particles as it can be seen from SEM images, where the size of the particles deposited by using only the photocatalysts were lower than that deposited by re-immersing the materials into polyacrylic binders. These findings are in accordance with other studies where the fabrics’ treatment with TiO_2_-acrylate revealed more agglomerated particles [34].

Physical characteristics (weight, thickness, fiber diameter, air permeability, water vapor resistance, and surface resistivity) were investigated initially and after the fabrics’ treatment to evaluate the effect of co-doped TiO_2_ nanoparticles on the properties of polyester material (Table 2). In addition, the percentage weights of Ti K are shown in Table 3.

As shown in Table 2, the results demonstrated that no significant changes were induced by the treatment, except air permeability, especially in the case of fabrics covered with photocatalyst 2 (HT2). This behavior can be attributed to the larger size of photocatalyst 2 nanoparticles (~15 nm) in comparison with photocatalyst 1 (~10 nm) which coated the fibers and penetrated the pores. Certainly, an important role in the decrease of air permeability was played by the polyacrylic binder due to the film formation on the material’s surface. The water vapor resistance showed only minor modifications, indicating that the comfort properties were conserved. The slight increase of the material weight (~5%) and thickness was due to the deposition of photocatalytic compounds and polyacrylic binder. The surface resistivity values indicated a higher increase for the fabrics covered with a polymeric film than for those treated only with nanoparticles.

Due to the interaction of negatively charged groups (–COO^−^) with the positive charges of photocatalysts, the polyacrylic binder improves the adherence of the particles at the material surface allowing the deposition of a much larger amount of photocatalysts on the polyester fabric. If we compare the amount of photocatalysts deposited by using the dispersion remaining from the first treatment (fabrics named HT1 ITO and HT2 ITO) with that obtained by using the initial dispersion (fabrics named HT1 and HT2), it is clear that the re-used dispersion in combination with polyacrylic binder almost tripled the quantities of particles deposited on the fabrics. As the results demonstrate, the amount of photocatalyst 2 deposited on fabrics was double in comparison with photocatalyst 1.

### 2.3. Evaluation of the Photocatalytic Efficiency

Photocatalytic activity assessed in terms of chromaticity coordinates of the treated materials, stained with methylene blue and coffee and exposed to light, is shown in Table 4.

The color differences between the treated samples and the control were measured via the CIE L*a*b* coordinates specified by the “Commission Internationale de l’Éclairage” (CIE) [35], where: L* represents lightness (dL* is the lightness difference between sample and control and indicates a lighter color if positive and darker if negative), a* signifies the red/green value (if da* is positive, the sample is redder than the control, otherwise is greener), and b* is the yellow/blue value (if db* is positive, the sample is yellower than the control, otherwise it is bluer), and dE* represents the total color difference and is calculated according to the following formula: dE* = $\sqrt{dL{}^{*2}+da{}^{*2}+db{}^{*2}}$.

As it can be seen in Table 4, the most efficient discoloration took place under visible and solar radiation for both types of stains. The improved photocatalytic activity could be attributed to the extended absorption [36] of the light due to the synergistic effect of Fe–N co-doping of TiO_2_ resulting in the formation of narrow band gap level, reducing the excitation energy of electron from the valence band to conduction band [37] and improving the generation of electron-hole pairs. Depending on synthesis technology the optical absorption edge is shifted to 675 nm [38].

**Figure 4:**
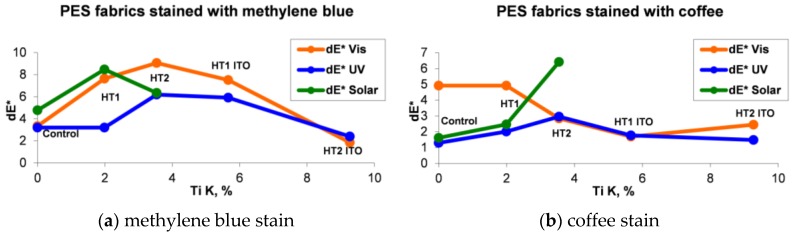
Correlation between percentage weights of Ti K and color differences (dE*) of the polyester fabrics treated with TiO_2_-1% Fe–N photocatalysts, stained with methylene blue (**a**) or coffee (**b**), and exposed to visible, ultraviolet (UV), and solar light.

The correlation between the percentage weights of Ti K and the color differences (dE*) of the polyester fabrics treated with TiO_2_-1% Fe–N photocatalysts and stained with methylene blue or coffee is shown in Figure 4. If under visible and UV light, the HT2 fabric stained with methylene blue and containing an average amount of TiO_2_ (3.54% Ti K) had the highest photocatalytic efficiency, under solar radiation, the highest activity was shown for the HT1 fabric containing the lowest amount of TiO_2_ (1.99% Ti K). In the case of the materials stained with coffee, the highest photocatalytic efficiency under visible light was shown by the HT1 material on which surface it was found 1.99% Ti K and, under UV and solar light, by the HT2 material containing 3.54% Ti K.

What is important to notice is that, no matter what type of light or stain that was used, the photocatalytic activity increases until a certain concentration of nanoparticles, after which the effect strongly decreases. These results confirm the findings of other studies [20] demonstrating that an increased concentration of photocatalyst [39] determines higher degradation rates, but the effect is diminished [40,41,42] by the particle size and agglomeration, and by the contaminant’s type and loading.

In the case of coffee stains, the efficiency was higher for the materials treated with photocatalysts 1 and 2, but 1 was more efficient compared to 2. This effect could be attributed to the smaller size of photocatalyst 1 (~10 nm), leading to a higher relative surface area and number of active sites.

The coffee stains are more resistant to light compared to methylene blue dye, the color differences indicating a discoloration similar to the untreated material for photocatalyst 1, and even a stabilization of color when treating with photocatalyst 2 (Table 3). The low ability of TiO_2_ to decompose coffee stains was reported by other studies, probably because of the aromatic rings of components [43] which are difficult to be mineralized.

More than that, as studies have shown, the photo-degradation mechanisms are different for dyes and for natural pigments [44]. While synthetic dyes are sensitive [45] to light, and the stain discoloration is initiated by the dye itself, the natural compounds, as those found in coffee, are decomposed by reactive species, such as –OH, O_2_ and H_2_O_2_ generated on the TiO_2_ surface [46]. Summarizing, the results demonstrate that the photocatalytic activity depends on multiple parameters, starting from the photocatalysts’ characteristics, to the type of additives and contaminants. It is clear that, for each parameter, many experiments have to be conducted to establish the optimum concentration at which the photocatalyst is efficient.

### 2.4. Antibacterial Efficiency

**Figure 5:**
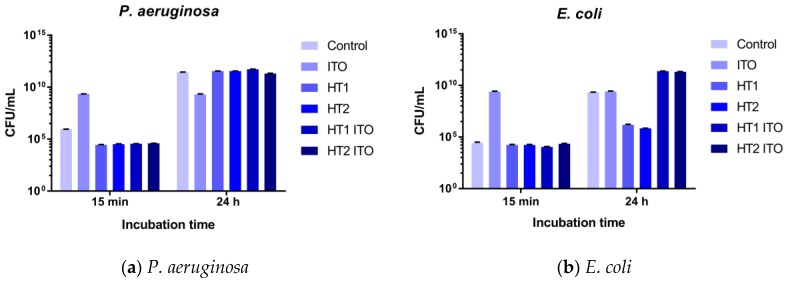
Graphic representation of CFU/mL of *P. aeruginosa* (**a**) and *E. coli* (**b**) viable cell counts recovered after 15 min and 24 h of contact with the photocatalyst-treated polyester fabrics.

The antibacterial activity against *E. coli* and *P. aeruginosa* strains and biocompatibility performances of the materials treated with TiO_2_-1% Fe–N particles were evaluated according to international standard methods. The two types of strains have been selected as the most frequent Gram-negative, fermentative and, respectively, non-fermentative bacterial species isolated from different types of nosocomial infections, i.e., urinary tract infections, pneumonia, blood stream infections, and surgical site infections [47]. The PES samples exhibited specific antimicrobial features, depending on the tested microbial strain and the incubation time (Figure 5). All particle-impregnated PES samples strongly inhibited the growth of *P. aeruginosa* after 15 min of incubation (*p* < 0.001; two-way analysis of variance, Bonferroni test), inducing more than 1 log decrease of viable cells counts. However, the inhibitory effect was not preserved after 24 h, excepting a very slight inhibitory effect of HT2 ITO. Interestingly, the ITO-treated PES material proved to be the most resistant to *P. aeruginosa* growth after 24 h of incubation, probably due to specific physicochemical features of this type of acrylic binder, interfering with the later phases of the biofilm’s development. In the case of the *E. coli* strain, although no significant inhibitory activity of samples treated with TiO_2_-1% Fe–N particles was noticed after 15 min of incubation, a very strong antibacterial activity was exhibited after 24 h by HT1 and HT2 fabrics, which induced a decrease of more than 2 logs in the number of colony forming units (CFU)/mL (Figure 5).

Taken together, the results regarding the antimicrobial efficiency of the treated fabrics revealed that all of these exhibited a more prominent antibacterial activity after 15 min of incubation against the *P. aeruginosa* strain. The HT1 and HT2 materials exhibited a very good antimicrobial activity against the *E. coli* strain after 24 h of incubation. The addition of the polyacrylic binder did not improve the antimicrobial activity of the obtained PES samples but, on the contrary, in some cases it seems to decrease it.

### 2.5. Biocompatibility Results

Before their use in developing novel textiles with improved antibacterial properties and self-cleaning capacity, these NP-treated PES samples must be strictly analyzed to check if they are biocompatible and safe for skin after direct contact or accidental release of nanoparticles from the fabric under the influence of various mechanical or chemical factors.

Therefore, to draw conclusions regarding the influence of these PES fabrics on the cytotoxicity, cell viability and cell membrane integrity, and their potential to generate inflammatory processes, multiple in vitro tests were performed on normal dermal fibroblasts. The exposure periods were selected at 6 h and 12 h, as the main time intervals at which toxic effects are visible on the skin during clothes wearing or after contact with such modified fabrics.

**Figure 6:**
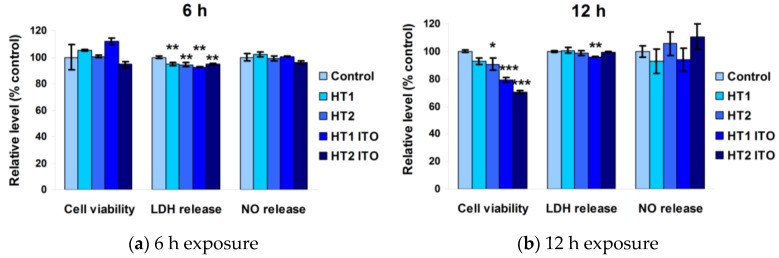
Biocompatibility of PES samples as shown by cell viability, lactate dehydrogenase (LDH), and nitric oxide (NO) release assays after 6 h (**a**) and 12 h (**b**) exposure on normal skin fibroblasts. Results are expressed as the mean ± standard deviation (SD) (*n* = 3) and represented relative to the untreated PES sample (control). *p < 0.05, **p < 0.01 and ***p < 0.001 compared to control.

At 6 h after dermal fibroblast exposure to modified PES samples, there were no significant changes in any of the analyzed parameters (Figure 6a). The HT1 ITO sample induced a slight increase in cell viability, suggesting that the proliferative capacity of the cells have not been disrupted at all in the presence of these fabrics. Additionally, nitric oxide (NO) and lactate dehydrogenase (LDH) levels were around the control values after 6 h of incubation, proving that NP-treated PES fabrics did not affect cell membrane integrity nor induce inflammatory processes.

The cell viability decreased by 30% only after 12 h of exposure and the amount of NO released in the culture medium registered as slightly increased by 10%, while the released LDH level remained unchanged compared to the control (Figure 6b). Therefore, NP-treated fabrics did not affect cell membrane integrity and the inflammatory processes induced as a response to these functional fabrics were too low to be considered significant.

**Figure 7:**
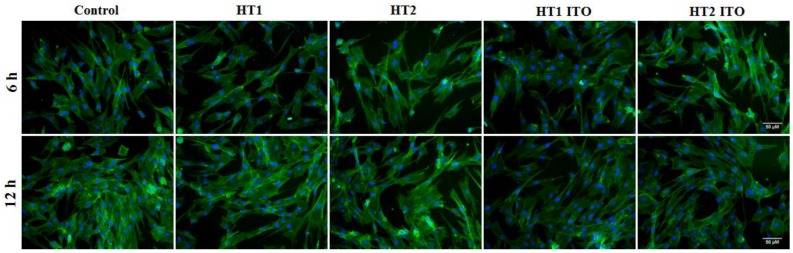
Actin cytoskeleton organization of dermal fibroblast cells after 6 h and 12 h of incubation with photocatalyst-treated PES samples. F-actin (**green**) was labeled with phalloidin-phalloidin-fluorescein isothiocyanate (FITC) and nuclei (**blue**) were counterstained with 4′,6-diamidino-2-phenylindole dihydrochloride (DAPI). Scale bar: 50 µm.

The actin cytoskeleton organization evidenced by fluorescence microscopy in Figure 7 was consistent with the results of biocompatibility tests showed in Figure 6. Thus, the cells kept their fibroblast-specific elongated morphology and established numerous focal adhesions after 6 and 12 h of incubation (Figure 7), which confirmed that NP-containing textiles did not affect the behavior of human dermal fibroblasts.

## 3. Discussion

Self-cleaning and self-sanitizing coatings containing titanium dioxide are often used to obtain biocompatible fabrics with antibacterial, antifungal, and antiviral activity that could find applications in the biomedical field [48,49,50]. In this context, the purpose of the present study was the design, manufacture, and bioevaluation of polyester fabrics impregnated with TiO_2_ particles co-doped with 1% of Fe and N, obtained under hydrothermal conditions at different pH values. The ability of different microorganisms to survive in environments characterized by strong selective pressures and their capacity to share genetic determinants, leading to increasing antimicrobial resistance, represent an important healthcare issue. The textile industry has produced antimicrobial fabrics by the addition of bactericidal compounds, such as nanoparticles, quaternary ammonia compounds, and broad-spectrum compounds, like triclosan [51]. The antimicrobial and antifungal properties of textiles treated with monometallic nanoparticles, such as copper and silver, are well established. In line with this, it has been shown that cotton and cotton/polyester textiles coated with Cu, Ag and Ti film produced total elimination of *E. coli* and *S. aureus* from the textile surfaces [52,53]. In our recent work, we characterized novel cotton fabrics coated with dispersions of nanoscaled TiO_2_-1% Fe–N particles prepared by the hydrothermal method which exhibited potent antimicrobial activity against *E. coli*, *P. aeruginosa*, and *S. aureus* [28]. In view of these results, we were able to develop in the current study novel polyester fabrics which were treated with photocatalytic TiO_2_ particles co-doped with iron and nitrogen atoms.

All treated fabrics described within this study inhibited the growth of Gram-negative, non-fermentative bacilli strains after 15 min of contact. These results demonstrated that the treated fabrics could decrease the risk of exogenous accidental contamination with *P. aeruginosa* in case of medical maneuvers with short duration, involving textiles, like changing dressings or wound washing. This could be a very significant aspect if we take into account that *P. aeruginosa* is a reputed nosocomial agent, with a very high resistance to different limitative conditions, and being very frequently transmitted in the hospital settings through contaminated materials, including textiles. After 24 h, the inhibitory effect of microbial growth was preserved only for the HT1 and HT2 samples against *E. coli*. However, the ITO-treated PES samples did not show the same efficiency. This could probably be explained by the nanoparticles’ agglomeration in large clusters and their covering with a polymer layer. Consequently, the number of the active sites generating reactive species involved in the photocatalytic process and the attack on bacterial cells were decreased. Therefore, further studies should be focused in finding novel ligands that could extend the duration of the antimicrobial activity of the modified textiles.

In addition to the fact that PES fabrics treated with photocatalytic TiO_2_-1% Fe–N nanoparticles could provide short-term protection against microbial colonization of these materials, these modified materials were harmless for skin cells, proving good biocompatibility. LDH and NO assays are two of the most common in vitro cytotoxicity tests. The release of intracellular LDH into the culture medium as a result of cell membrane damages represents a valuable marker for cell death [54]. As long as the LDH leakage level is maintained under the 10% threshold, the effect of photocatalyst exposure can be considered insignificant for membrane toxicity [55]. Further, it is well known that NO is an important molecule involved in inflammatory responses, but it also plays a key role in the regulation of apoptotic death and cell viability. The influence of NO on cell viability is concentration- and cell type-dependent. It appears that higher levels induce apoptotic death, while lower or moderate concentrations protect the cells, favoring their survival [56]. Most of the previous studies have addressed the photocatalytic performance of the textiles with TiO_2_ coatings for self-cleaning or purification applications, but did not focus on the critical aspect of human cytotoxicity [57,58]. Thus, it is difficult to compare our findings, but we can state that they are in agreement with previous safety profiles revealed on macrophage, liver, and kidney cells for different prepared photocatalytic TiO_2_ nanoparticles and loaded on the surface of polyester-cotton fibers [59].

Taken together, we show that the treatment of PES fabrics with photocatalytic TiO_2_-1% Fe–N nanoparticles could provide novel functionalized textiles harboring antibacterial and cytocompatible properties. Thus, our results highlight the potential of these modified fabrics for the development of novel materials for biomedical applications, such as wound dressings, disposable operatory fields, hospital sheets, and medical robes for preventing patients’ accidental contamination with microorganisms from the hospital environment.

## 4. Materials and Methods

### 4.1. Synthesis and Characterization of Photocatalysts

The TiO_2_-1% Fe–N (titanium dioxide doped with 1% iron atoms and nitrogen) photocatalysts were synthesized hydrothermally at 200 °C for 2 h, starting with corresponding amounts of FeCl_3_·6H_2_O and TiCl_3_. A solution of 25% NH_4_OH was used to adjust the pH values to 8.5 for photocatalyst 1 and 5.5 for photocatalyst 2. The resulting powders were dried in air at 105 °C and finally calcined at 400 °C in air for 2 h. The complete procedure was described in more details in a previous paper [28]. TEM investigations have been performed using a JEOL JEM ARM200F-Tokyo-Japan transmission electron microscope operating at 200 kV on specimens prepared by crushing the powders in ethanol, dispersing by sonication, and dropping on lacy carbon grids.

The method to prepare the 0.5 g/L photocatalyst dispersions and to deposit them on fabrics was previously defined by the authors [28]. Shortly, 0.2 g photocatalyst powder and 0.006 g polyvinylpyrrolidone were dispersed in 400 mL of distilled water in an ultrasonic bath at 40 °C for 60 min. One hundred percent polyester woven fabric (146.30 g/m^2^, 0.39 µm thickness) was immersed in the 0.5 g/L photocatalyst dispersion, sonicated for 60 min at 40 °C, and then dried in an oven at 100 °C for 60 min. A second fabric was immersed in the used dispersion and treated to similar conditions as above. To fix the particles, the wet material was immersed in 20 mL/L polyacrylic binder (ITOBINDER AG, abbreviated ITO in the present work, it is a self-cross linking aqueous acrylic copolymer emulsion acquired from LJ Specialties, Chesterfield, Derbyshire, UK), maintained in an ultrasound bath for 30 min, and then dried at 100 °C.

The treated fabrics were noted: HT1-PES fabric treated with TiO_2_-1% Fe–N (photocatalyst 1), HT2–PES fabric treated with TiO_2_-1% Fe–N (photocatalyst 2); HT1 ITO-PES fabric treated with TiO_2_-1% Fe–N (photocatalyst 1) and ITO; HT2 ITO-PES fabric treated with TiO_2_-1% Fe–N (photocatalyst 2) and ITO; and control PES—untreated PES fabric.

The morphology of the fabrics’ surfaces treated with photocatalysts and the amount of deposited particles were analyzed by SEM (Quanta 200, FEI, Eindhoven, The Netherlands) equipped with an EDX detector, which was used to evaluate the elemental composition of the coated textiles. EDX spectrum measurements were performed on fabric samples attached to a carbon conductive tape with 10 kV accelerating voltage. The spectral data were collected from an area of 0.0379 mm × 0.0296 mm with 512 × 400 resolution, at 8000× magnification. After ZAF (the acronym for atomic number effects, absorption and fluorescence) correction coefficients, the atomic and mass ratios of titanium were calculated using the software Genesis Imaging/Mapping 5.21.

Evaluation of the photocatalytic performance was performed by staining the control materials and those treated with photocatalysts with a 0.01 g/L MB solution and coffee (three teaspoons of coffee in 500 mL water). The stained fabrics were half covered with paper and were exposed to UV light (254 nm) in a completely closed cabinet and in a visible light laboratory equipment (Xenotest, 1000 W xenon arc lamp, irradiance 4.5 mW/cm^2^ at 300–400 nm, Heraeus Industrietechnik, Hanau, Germany). Additionally, the fabrics were exposed to natural solar radiation. Chromaticity coordinates of exposed and non-exposed material were measured by a UV-Vis Hunterlab spectrophotometer (Hunter Associates Laboratory, Reston, VA, USA), with a CIELAB 1976 color space and D65-light source.

### 4.2. Antimicrobial Activity Assay

The antimicrobial activity of the obtained textiles was assessed against Gram-negative bacterial reference strains, i.e., *P. aeruginosa* ATCC 27853 and *E. coli* ATCC 8739. The microbial strains were purchased from the American Type Culture Collection (ATCC, Manassas, VA, USA). Glycerol stocks were streaked on Luria Bertani (LB) agar in order to obtain 24 h cultures for further experiments. The biofilm development was assessed after two incubation times, i.e., 15 min and 24 h, in accordance with the ASTME 2149-10 standard for the assessment of the antimicrobial activity of agents immobilized in dynamic testing conditions. For this purpose, the textile materials were cut into equal circular pieces of 8 mm diameter and sterilized by autoclaving at 121 °C for 15 min. The sterile pieces were then immersed in one mL of microbial suspensions of ~10^7^ CFU/mL performed in sterile saline and left in contact for 15 min and 24 h. At the end of exposure, the microbial suspensions incubated with the tested samples were vortexed and 10 µL of serial ten-fold dilutions of the obtained suspension were plated in triplicate on LB agar. After 24 h of incubation at 37 °C, viable cell counts were performed and the CFU/mL values for each sample were established.

### 4.3. In Vitro Biocompatibility Assessment

CCD-1070Sk normal human skin fibroblasts (purchased from American Type Culture Collection (ATCC), Cat. No. CRL-2091, Rockville, MD, USA) were cultured at low passage in complete Eagle’s minimum essential medium (MEM; Gibco/Invitrogen, Carlsbad, CA, USA) containing 10% fetal bovine serum (FBS; Gibco/Invitrogen, Carlsbad, CA, USA) at 37 °C in a humidified atmosphere with 5% CO_2_. Fibroblasts were grown to 70%–80% confluence within five days, then detached with 0.25% trypsin—0.03% ethylenediaminetetraacetic acid (EDTA) and transferred to new culture flasks. For biocompatibility assessment, the cells were seeded at a density of 2 × 10^4^ cells/cm^2^ in a 24-well plate and left to adhere overnight. The polyester fabrics treated with TiO_2_-1% Fe–N samples, cut into 1 × 1 cm^2^ pieces, sterilized at 120 °C for 20 min, and exposed to a light source for 30 min were soaked in culture medium and placed over the attached fibroblasts without disturbing the cells as it was previously reported for other materials [48]. After 6 and 12 h of incubation, cell morphology and viability were evaluated, while cytotoxicity tests were also performed. The results were expressed relative to the untreated PES fabric used as a control.

The cell viability was measured using the 3-(4,5-dimethylthiazol-2-yl)-2,5-diphenyltetrazolium bromide (MTT; Sigma-Aldrich, St. Louis, MO, USA) assay (from the battery of cytotoxicity tests described in ISO 10993-5:2009, part 5) which is based on the quantification of NAD(P)H-dependent cellular oxido-reductase enzymes activity in the viable cells. Briefly, the culture medium and polyester samples were removed at the end of the time exposure and the cells were incubated with 1 mg/mL MTT solution for 3 h at 37 °C and 5% CO_2_. The purple formazan crystals formed in the viable cells were dissolved with 2-propanol (Sigma-Aldrich, St. Louis, MO, USA) and the absorbance was measured at 595 nm using a microplate reader (GENios Tecan, Grödig, Austria).

The LDH amount released in culture medium was assessed as a measure of cell membrane integrity using a commercial kit (TOX7, Sigma-Aldrich, St. Louis, MO, USA) according to the manufacturer’s instructions. Volumes of 50 µL of culture supernatants were incubated with a 100 µL mix composed from equal parts of dye, substrate, and cofactor for 30 min in dark. The reaction was stopped by adding 15 µL of 1N HCl and the absorbance was read at 490 nm using a GENios Tecan microplate reader (Tecan, Grödig, Austria).

The level of NO released in the culture medium as an indicator of inflammation was determined using the Griess reagent. Culture supernatants were mixed with an equal volume of Griess reagent, which is a stoichiometric solution (*v*/*v*) of 0.1% naphthylethylendiamine dihydrochloride and 1% sulphanilamide in 5% H_3_PO_4_, and absorbance was read at 550 nm using a GENios Tecan microplate reader. NO concentration was extrapolated on a NaNO_2_ standard curve.

Cell spreading and actin cytoskeleton morphology were monitored via fluorescence imaging using cells fixed with 4% paraformaldehyde for 20 min and permeabilized with 0.1% Triton X-100—2% bovine serum albumin for 1 h. Filamentous actin (F-actin) was labeled with 20 µg/mL phalloidin conjugated with FITC (Sigma-Aldrich, Munich, Germany) and nuclei were stained with 2 µg/mL 4′,6-diamidino-2-phenylindole (DAPI) (Molecular Probes, Life Technologies, Carlsbad, CA, USA). Images were captured using an inverse fluorescence microscope Olympus IX71 (Olympus, Tokyo, Japan).

### 4.4. Statistical Analysis

The antibacterial efficiency and cell culture assays were performed in triplicate, and data were shown as mean ± SD. The statistical significance was analyzed by Student’s *t*-test or two-way ANOVA followed by Bonferroni’s post hoc test using GraphPad Prism 5 (GraphPad software Inc., La Jolla, CA, USA), and a value of *p* < 0.05 was considered significant.

## 5. Conclusions

In this work, we revealed that the physical properties of the polyester fabrics treated with nitrogen and iron co-doped TiO_2_ nanoparticles prepared by hydrothermal method were not significantly modified. Also, the use of a polyacrylic binder allowed the deposition of a higher amount of nanoparticles on the textile surface and the improvement of the photo-discoloration efficiency under visible and solar radiation due to the extended absorption of light in the visible area. Finally, our results highlighted the biocompatibility on human skin cells of PES fabrics modified with photocatalytic TiO_2_-1% Fe–N nanoparticles which could be further used as novel materials for biomedical applications, such as wound dressings, disposable operatory fields, hospital sheets, and medical robes for preventing patients.

## Figures and Tables

**Table 1:** Phase content and crystallite size of hydrothermally-synthesized photocatalysts.

Sample	Crystallite Size (nm)	Phase Assignment/Abundance (wt %)
(Photocatalyst 1)	12.3	Anatase 85.3
	8.5	Brookite 14.7
(Photocatalyst 2)	10.4	Anatase 79.4
	11.6	Brookite 20.6
Errors	±1.5	±1.4

**Table 2:** Physical characteristics of initial and coated polyester fabrics.

Parameters	Results for Each Sample					Standard
	Initial	HT1	HT1 ITO	HT2	HT2 ITO	
Weight (g/m^2^)	142	149.65	150	150.55	150	SR EN ISO 12127/2003
Thickness (mm) (surface: 20 cm^2^, ∆*pres*: 100 Pa/200 Pa, *t*: 19.2 °C, *RH*: 63.2%)	0.295	0.36	0.36	0.39	0.36	SR EN ISO 5084/2001
Fiber diameter (μm)	11.69	11.13	10.72	11.33	11.18	Projection microscope
Air permeability (L/m^2^/s) (20 cm^2^, 100 Pa, *t*: 19.2 °C, *RH*: 63.2%)	173.3	153	106.33	86.33	99.86	SR EN ISO9237:1999
Water vapor resistance (m^2^Pa/W)	6.519	6.439	6.679	5.999	6.678	SR EN 31092/A1/2013 ISO 11092 /1997
Surface resistivity (×10^14^ Ω) (*t*: 24.1 °C, *RH*: 45.4%)	1.75	2.2	2.6	1.3	2.7	SR EN 1149-1:2006

Notes: *t* is temperature; *pres*, pressure; *RH*, relative humidity; HT1, polyester fabric treated with photocatalyst 1; ITO, polyacrylic binder; HT2, polyester fabric treated with photocatalyst 2; SR EN ISO, European Standard of the International Organization for Standardization adopted as Romanian Standard.

**Table 3:** Energy-dispersive X-ray spectroscopy (EDX) analysis of polyester fabrics treated with TiO_2_-1% Fe–N photocatalysts.

Element (wt %)	Control	HT1	HT1 ITO	HT2	HT2 ITO
C K	61.51	65.43	58.57	62.12	53.74
O K	38.49	32.58	35.78	34.34	36.99
Ti K	0	1.99	5.66	3.54	9.27
Total	100	100	100	100	100

**Table 4:** Color differences (dE*) of the polyester fabrics treated with TiO_2_-1% Fe–N photocatalysts, stained with 0.01 g/L methylene blue (MB) or coffee, and exposed to visible, ultraviolet (UV) and solar light.

Sample	Stained with Methylene Blue			Stained with Coffee		
	dE* Vis	dE* UV	dE* Solar	dE* Vis	dE* UV	dE* Solar
Control	3.34	3.21	4.77	4.92	1.3	1.62
HT1	7.64	3.21	8.47	4.92	2.01	2.47
HT2	9.06	6.2	6.35	2.84	2.96	6.42
HT1 ITO	7.51	5.91		1.69	1.77	
HT2 ITO	1.86	2.4		2.45	1.48	
